# The Influence of [110] Compressive Stress on Kinetically Arrested B2–R Transformation in Single-Crystalline Ti–44Ni–6Fe and Ti–42Ni–8Fe Shape-Memory Alloys

**DOI:** 10.3390/ma17010051

**Published:** 2023-12-22

**Authors:** Mitsuharu Todai, Takashi Fukuda, Tomoyuki Kakeshita

**Affiliations:** 1 Department of Environmental Materials Engineering, National Institute of Technology, Niihama College, 7-1 Yagumo-cho, Niihama 792-8580, Ehime, Japan; 2 Institute of Industrial Science, The University of Tokyo, 4-6-1, Komaba Megro-ku, Tokyo 153-8505, Japan; 3 Department of Materials Science and Engineering, Graduate School of Engineering, Osaka University, 2-1, Yamada-oka, Suita 565-0871, Osaka, Japan; fukuda@mat.eng.osaka-u.ac.jp (T.F.); kakeshita@mat.eng.osaka-u.ac.jp (T.K.); 4 Department of Mechanical Engineering, Faculty of Engineering, Fukui University of Technology, 3-6-1, Gakuen, Fukui 910-0028, Fukui, Japan

**Keywords:** kinetics, shape-memory alloys, titanium–nickel alloys, thermal arrest, isothermal transformation, elastic constants

## Abstract

Ti–(50−*x*)Ni–*x*Fe alloys exhibit a thermally induced B2–R martensitic transformation (MT) when *x* is between 1.5% and 5.7%, whereas this transformation is suppressed when *x* is 6 at% and higher. We studied the reason for this suppression by applying compressive stress in the [110]_B2_ direction to single-crystalline Ti–44Ni–6Fe and Ti–42Ni–8Fe (at%) alloys. Under stress, these alloys exhibit a B2–R MT with a large temperature hysteresis of ≥50 K. The B2–R MT in these alloys is probably thermally arrested, and a small entropy change is a possible reason for this arrest. The Young’s modulus *E*_[110]_ of these alloys significantly decreases with decreasing temperature, and the B2–R MT under stress occurs at a temperature where *E*_[110]_ is approximately 50 GPa. Presumably, lattice softening assists the B2–R MT.

## 1. Introduction

Martensitic transformations (MTs) are classified into two groups based on kinetics [1]. One is athermal MTs, in which the martensite phase fraction depends on temperature but not time. The other is isothermal MTs, in which the martensite phase fraction depends on both the temperature and time. Thermoelastic MTs in shape-memory alloys (SMAs) are typically considered thermal MTs. However, recent studies have revealed that isothermal MTs occur in many shape-memory alloys (SMAs) that exhibit athermal MTs. The isothermal nature of thermoelastic MTs is particularly discussed in Cu-based SMAs [2], Ni–Mn based SMAs [3,4,5], Ti based alloys [6,7,8], and Ti–Ni-based SMAs [9,10,11,12,13,14].

Isothermal MTs proceed via thermal activation processes such as diffusional transformations. To form the martensite phase, potential barriers must be overcome via thermal activation. Therefore, the transformation behavior depends on the relationship between the height of the potential barriers and the thermal energy *k*_B_*T*. If the thermal energy is sufficiently high to overcome the potential barrier, MT will occur in a short period, and decreasing the isothermal nature of MT using conventional experiments is difficult. However, if the thermal energy is much smaller than the height of the potential barrier, MT cannot occur on a conventional time scale, even if the free energy of the martensite phase is lower than that of the parent phase. In this case, we believe that the MT is kinetically arrested. Otherwise, if the thermal energy is of the same order as the height of the potential barriers, the potential barriers will be overcome after several trials by thermal activation processes, and the MT will show a clear time dependence on the conventional time scale. We may consider that the MT in an alloy is kinetically arrested when the temperature is between the equilibrium temperature *T*_0_ and the martensitic transformation start temperature *M*_s_. In this temperature range, the MT may occur after a sufficiently long time.

Isothermal holding experiments at temperatures between *T*_0_ and *M*_s_ effectively revealed the isothermal nature of the MTs. A drastic change in the electrical resistivity while holding above *M*_s_ has been reported for Cu–Al–Ni [2] and Fe–Ni alloys [2]. Considering these reports, Ren et al. examined isothermal holding experiments using a Ti–50at%Ni alloy and reported that the resistivity did not change when holding above its *M*_s_ temperature [15]. Based on these results, they concluded that Ti–Ni alloys do not exhibit isothermal MT behavior.

Kustov et al. [10] revealed the isothermal nature of MT in Ti–Ni alloys. They performed isothermal holding experiments at intermediate temperatures between *M*_s_ and *M*_f_ (the martensitic transformation finish temperature). They observed a notable change in electrical resistivity while maintaining these temperatures. The isothermal nature of MT in Ti–Ni alloys was confirmed by other researchers [11,12,13,14]. Moreover, the time dependence of the fraction of the B19′ martensite phase was systematically studied [12,13].

Most reports on isothermal MT in Ti–Ni alloys are related to the B2-B19′ or R-B19′ martensitic transformations [9,10,11,15]. Concerning the B2–R MT, Kustov et al. considered this transformation athermal, while the B2-B19′ and R-B19′ transformations showed an isothermal nature [9]. By contrast, we observed an isothermal B2–R MT in a Ti–44Ni–6Fe alloy [16]. Before describing this behavior, we shall briefly summarize the B2–R MT in Ti–Ni alloys.

The B2–R MT in Ti–Ni alloys is characterized by a small temperature hysteresis of 5 K or less [17,18]. The R-phase exhibits a trigonal structure with the space group P3 [19] or P$\bar{3}$ [20]. Because the c-axis of the R-phase corresponds to one of the <111>_B2_ directions, four corresponding variants of the R-phase are formed in the specimen [19,21]. The R-phase expands in the <111>_B2_ direction and contracts perpendicularly. The B2–R MT is a first-order martensitic transformation that proceeds via a nucleation and growth mechanism [22]. The B2–R MT is preceded by the phonon softening of the TA_2_ branch and the appearance of diffuse satellites in the <110>*_B2_ direction [23,24,25,26,27,28,29,30] (here, the asterisks indicate the reciprocal direction). These are referred to as precursor phenomena.

**Figure 1:**
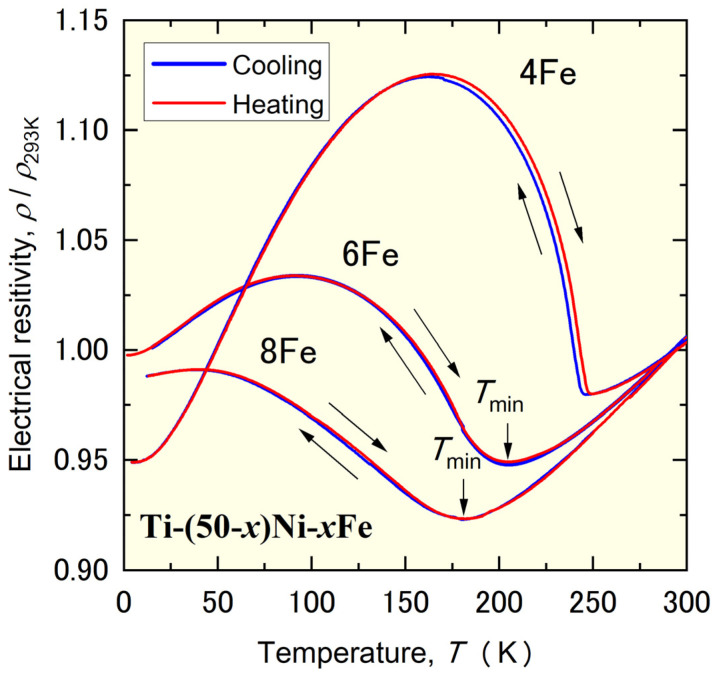
Temperature dependence of the electrical resistivity of Ti–46Ni–4Fe, Ti–44Ni–6Fe, and Ti–42Ni–8Fe alloys reproduced from Ref. [28].

Choi et al. systematically studied the B2–R MT using a series of Ti–(50−x)Ni–xFe alloys [28]. (We refer to each alloy according to its Fe content; for example, the 6Fe alloy.) They found that the B2–R transformation temperature decreased linearly as the Fe content increased up to 5.7%, but the transformation was suppressed in alloys containing 6 at% Fe and more. Figure 1 shows the temperature dependence of the electrical resistivity of the 4Fe, 6Fe, and 8Fe alloys [28]. The sharp increase in the resistivity and hysteresis between the cooling and heating processes caused by the B2–R MT in the 4Fe alloy did not appear in the 6Fe and 8Fe alloys. They also reported that these alloys exhibited diffuse satellites in their electron diffraction patterns below the temperature at which the resistivity exhibited a local minimum (*T*_min_). The diffuse satellites moved from an incommensurate position to a commensurate position for the R-phase in the 6Fe alloy, whereas they remained in the incommensurate position in the 8Fe alloy. Thus, the 6Fe alloy exhibited a B2-IC-C phase transformation, whereas the 8Fe alloy only showed a B2-IC phase transformation. Diffuse satellites are related to the formation of the nano-sized domain [24,25,26,27,28], and this state is frequently referred to as the strain-glass state [31,32,33,34,35].

Recently, similar anomalies in the temperature dependence of electrical resistivity and diffuse satellites at incommensurate positions have been reported not only in the B2–R MT in Ti–(50−x)Ni–xFe alloys but also in the B2–R MT of Ti_(50−x)_Ni_(47+x)_Fe_3_ alloys [36], B2-B19′ MT of Ti–Ni binary alloys [37], and B2-B19 MT of Cu-doped Ti-Ni alloys [38].

Here, we discuss the aforementioned isothermal B2–R MT method. Because the temperature hysteresis of the B2–R MT is usually smaller than 5 K, it is difficult to reveal an isothermal B2–R MT by holding it between *T*_0_ and *M*_s_. We considered the fact that the B2–R transformation is kinetically arrested in the 6Fe alloy, and isothermal holding for a prolonged period may induce it. We detected isothermal B2–R MT in the 6Fe alloy while holding it at 150 K for 50 h through electrical resistivity measurements [16].

If the B2–R MT in the 6Fe and 8Fe alloys is kinetically arrested, the MT is easily induced by external stress. Because the R-phase expands in the <111>_B2_ direction and contracts perpendicular to it, the application of tensile stress in the [111]_B2_ direction or compressive stress in the [110]_B2_ direction will assist the B2–R MT. As a preliminary experiment, we applied compressive stress in the [110]_B2_ of the 8Fe alloy and detected an MT [39]. We also applied tensile stress in the [111]_B2_ direction of 6Fe and measured the MT [40]. In both the alloys, the product phase formed by MT was most likely the R-phase. However, the transformation behavior of the 8Fe alloy under [110]_B2_ compressive stress was completely different from that of the 6Fe alloy under [111]_B2_ tensile stress.

One important difference was the temperature hysteresis of the MT. The temperature hysteresis observed in the 8Fe alloy under compressive stress was more than 50 K, whereas that in the 6Fe alloy under tensile stress was smaller than 1 K. Another important difference was the sharpness of the MT. While the 8Fe alloy showed a sharp transformation strain near *M*_s_, the 6Fe alloy showed a gradual transformation strain over a wide temperature range. There are two possible reasons for this discrepancy. The first reason is the difference in the orientation of the stress; the B2–R transformation behavior under [110]_B2_ compressive stress could be completely different from that under [111]_B2_ tensile stress when the transformation is kinetically arrested. Another possible reason is the difference in the parent phase state; the diffuse satellites appear at the incommensurate position (IC-phase) in the 8Fe alloy, whereas they appear at the commensurate position (C-phase) in the 6Fe alloy. This difference in the crystal structure of the parent phase may affect the transformation behavior.

To clarify the cause of the different behaviors of the B2–R MT, we need to examine the MT either (i) under [110]_B2_ compressive stress for both the 6Fe and 8Fe alloys or (ii) under [111]_B2_ tensile stress for both the 6Fe and 8Fe alloys. In this current study, we systematically investigated MT under [110]_B2_ compressive stress for both 6Fe and 8Fe alloys. During the B2–R MT, the B2 lattice stretched to the <111>_B2_ orientation, as described above. When the B2-phase was subjected to tensile stress in the [111]_B2_ direction, one variant tended to grow. However, when compressive stress was applied to the B2-phase in the [110]_B2_ direction, two variants perpendicular to this orientation tended to grow, suggesting a difference in the effect of self-accommodation. In this study, we confirmed that the B2–R MT behavior in 6Fe and 8Fe alloys under compressive stress was essentially the same, exhibiting a large hysteresis. We then proposed the reason as to why a large hysteresis appears for the B2–R MT. In addition, the role of lattice softening in the B2–R MT is discussed. Recently, a relationship between stress and magnetic transition was reported [41]; however, it was not considered in this study.

## 2. Materials and Methods

Ti–44Ni–6Fe and Ti–42Ni–8Fe (at%) alloys were prepared in an arc melting furnace (ACM-S01, DIAVAC, Chiba, Japan) using sponge Ti (99.9 mass%), electrolytic Ni (99.9 mass%), and electrolytic Fe (99.9 mass%) as the starting materials. Single-crystalline rods of these alloys were grown by the floating zone apparatus, which is made by Crystal Systems Corp. with a growth rate of 10 mm/h^−1^. The rods were homogenized at 1273 K for 24 h and then quenched in ice water. We first determined the electrical resistivity of the single crystals using the physical property measurement system (PPMS, Quantum Design, San Diego, CA, USA). Thus, we confirmed that both crystals showed identical behavior, as shown in Figure 1. Hereafter, we refer to the Ti–44Ni–6Fe alloy as the 6Fe alloy and the Ti–42Ni–8Fe alloy as the 8Fe alloy. Parallelepiped specimens with three edges parallel to the (001)_B2_, ($\bar{1}$10)_B2_, and (110)_B2_ planes were cut from each crystal using an electrical discharged machine (CONT HS-300, Brother, Kariya, Japan). The lengths of the three edges of the 6Fe alloy were 2.7, 2.6, and 8.5 mm; those of the 8Fe alloy were 2.4, 3.6, and 10.5 mm. These specimens were subjected to thermal expansion measurements under compressive stress, compressive tests at fixed temperatures using a Shimadzu autograph, and X-ray measurements under compressive stress. To evaluate the elastic constants, we also cut a parallelepiped specimen with three edges parallel to (100)_B2_, (010)_B2_, and (001)_B2_ from the 6Fe alloy. The size of this specimen was 2.85 mm × 3.42 mm × 3.93 mm. After cutting these shapes, all specimens were heat-treated at 1273 K for 1 h followed by quenching in ice water.

The thermal expansion and stress–strain curves were measured by applying a compressive stress in the [110]_B2_ direction. The strain of the specimen was evaluated using a strain gauge (KFL-02-120-C1-11), calibrated using a quartz plate. The elastic constants were evaluated using the rectangular parallelepiped resonance (RPR) method by using the physical property measurement system (PPMS, Quantum Design, San Diego, CA, USA). X-ray experiments under compressive stress were performed using Cu Kα radiation. Cooling-stage measurements were obtained by using MAC Science MXP3.

## 3. Results and Discussion

### 3.1. Martensite Phase Formed under Compressive Stress

**Figure 2:**
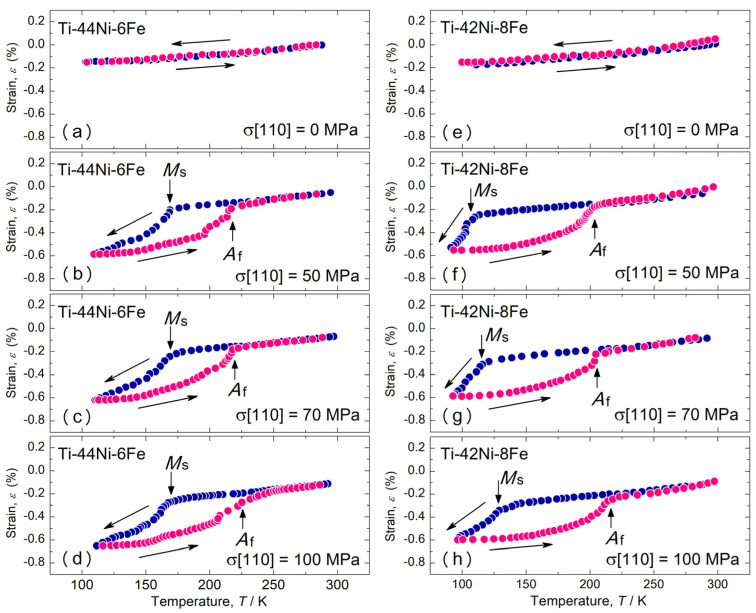
Thermal expansion in the [110]_B2_ direction of the Ti-44Ni-6Fe (**a**–**d**) and Ti-42Ni-8Fe (**e**–**h**) alloys measured in the cooling process and subsequent heating process under various stresses. Measurements were made under compressive stress applied in the [110]_B2_ direction.

We first describe the thermally induced MT behavior under compressive stress. Figure 2 shows the thermal expansion in the [110]_B2_ direction of the 6Fe and 8Fe alloys measured during the cooling and the subsequent heating processes under various compressive stresses applied in the [110]_B2_ direction. Under zero stress, both specimens contracted linearly during cooling, and no hysteresis occurred between the cooling and heating. This result is consistent with a previous report [39] that concluded that these alloys did not exhibit thermally induced martensitic transformations. The linear thermal expansion coefficient α was 1.0 × 10^−5^/K for both alloys, which is consistent with those obtained from the lattice parameters in a previous study [28]. Under a compressive stress of 50 MPa, both specimens exhibited a sudden decrease in strain during the cooling process, as indicated by the downward arrows. This sudden decrease was due to the B2–R MT. Therefore, we considered the temperatures indicated by the downward arrows as the MT start temperature (*M*_s_). The strain nearly completely recovered during the heating process at the temperatures indicated by the upward arrows; therefore, we consider these temperatures to be the reverse MT finish temperatures (*A*_f_). There was a large temperature hysteresis of over 50 K between the cooling and heating processes for both specimens. Similar results were obtained under stresses of 70 MPa and 100 MPa, as shown in Figure 2. The difference in the strain between the cooling and heating curves can be regarded as the transformation strain. This was approximately 0.3% for both the specimens.

**Figure 3:**
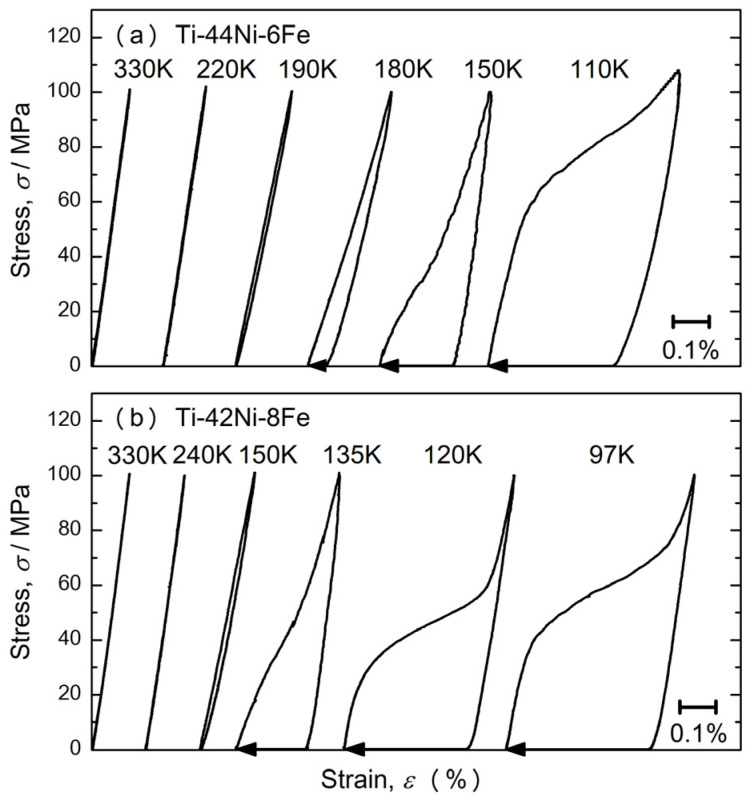
Stress–strain curve of the Ti–44Ni–6Fe (**a**) and Ti–42Ni–8Fe (**b**) alloys. Measurements were performed after cooling from 300 K to the test temperature under zero stress.

Next, we describe the stress-induced MT behavior at fixed temperatures. Figure 3 shows the stress–strain curves of the 6Fe (a) and 8Fe (b) alloys tested at various temperatures. At 330 K, the strain increased linearly during stress application and recovered completely during stress removal for both specimens. No hysteresis was detected between the stress application and removal processes. This implies that stress-induced MT does not occur at 330 K under compressive stresses of up to 100 MPa. The stress–strain curves of the 6Fe (8Fe) alloy exhibited hysteresis at temperatures of 190 K (150 K) and below. Although the strain completely recovered during the stress removal process at 190 K (6Fe) and 150 K (8Fe), residual strain appeared below these temperatures. The residual strain completely recovered during heating to 300 K, as indicated by the leftward arrows. The recovery of the strain implies that the hysteresis shown in Figure 3 is due to stress-induced MT. For both specimens, the hysteresis in the strain between the stress application and removal processes was approximately 0.3%, which coincides with the strain hysteresis in Figure 2. This agreement in the transformation strain implies that the stress-induced martensite phase is identical to the thermally induced martensite phase under compressive stress.

**Figure 4:**
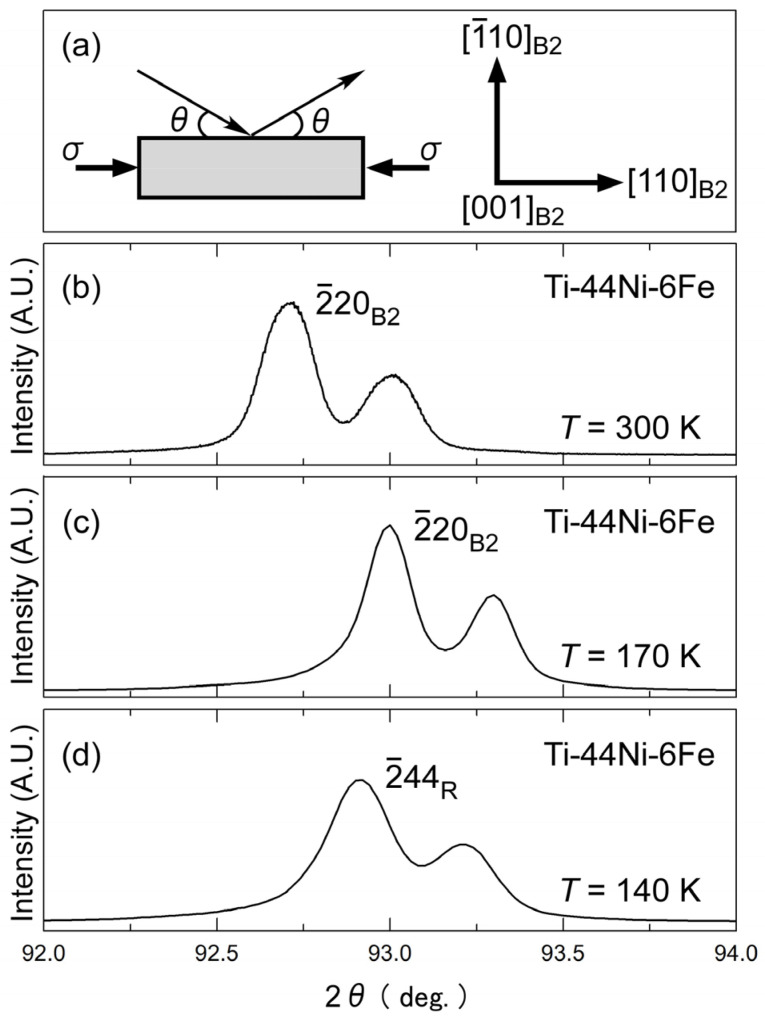
Experimental setup for X-ray diffraction under a compressive stress of 100 MPa applied in the [110]_B2_ direction (**a**) and X-ray profiles at 300 K (**b**), 170 K (**c**), and 140 K (**d**) obtained in the cooling process.

X-ray diffraction (XRD) was performed to understand the structure of the martensite phase formed under compressive stress. The experimental setup is shown in Figure 4a The specimen was compressed in the [110]_B2_ direction, and the scattering vector was set to normal to the ($\bar{1}$10)_B2_ plane. Figure 4b–d show the X-ray profiles of the 6Fe alloy under a compressive stress of 100 MPa obtained during successive cooling processes at 300, 170, and 140 K. The temperature of 170 K was slightly higher than *M*_s_, as shown in Figure 2. At each temperature, the profile was composed of two peaks: one from the Kα_1_ radiation and the other from the Kα_2_ radiation of the same plane. The *d*-values evaluated from the peak position in Figure 4 are 0.10644 nm at 300 K, 0.10619 nm at 170 K, and 0.10626 nm at 140 K. The peaks at 300 and 170 K can be regarded as the ($\bar{2}$20)_B2_ reflections, whereas the *d*-value at 140 K cannot be explained by $d_{\bar{2}20B2}$ because it is larger than the value at 170 K. If we extrapolate the temperature dependence of $d_{\bar{2}20B2}$ between 300 and 170 K to 140 K, $d_{\bar{2}20B2}$ at 140 K is expected to be 0.10613 nm. The experimental value at 140 K was 0.12% higher than that expected from the B2-phase. This peak was most likely the ($\bar{2}$44)_R_ reflection of the R-phase.

Incidentally, the (600)_R_ reflection, which was expected from the split of the ($\bar{2}$20)_B2_ reflection, was missing, possibly because of the lack of a corresponding R-phase variant. These variants expanded in the stress direction; therefore, they were not preferred under compressive stress. It is possible that the peak separation of the ($\bar{2}$44)_R_ and (600)_R_ in the 6Fe alloy was very small, which is another reason why a distinct (600)_R_ peak was not detected. The full width at half maximum (FWHM) increased at 140 K compared to the ($\bar{2}$20)_B2_ peak at 170 K. For the 8Fe alloy under compressive stress, we detected peak separation of ($\bar{2}$20)_B2_ into ($\bar{2}$24)_R_ and (600)_R_, as reported previously [39]. From the peak positions, the lattice parameters at 100 K were *a*_h_ = 0.737 nm and *c*_h_ = 0.523 nm. The hexagonal distortion, which is defined as $\eta =\frac{\sqrt{{2c}_{h}}}{a_{h}}-1,$ was obtained as 3.6 × 10^−3^ [39]. This value is of the same order as that of the R-phase (6.0 × 10^−3^) in the Ti–45Ni–5Fe alloy at 225 K [28].

As shown in Figure 2, the B2–R MT is suppressed in the 6Fe and the 8Fe alloys when an external stress is not applied. However, the specimens exhibited a stress-induced MT under a compressive stress of 50 MPa and were higher applied in the [110]_B2_ direction. The X-ray results shown in Figure 4 suggest that the martensite phase formed in this process is the R-phase, but the temperature hysteresis in Figure 2 and the stress hysteresis in Figure 3 are more than ten times larger than those for the conventional B2–R MT. This raises the question as to whether the product phase is truly the R-phase. If it is the R-phase, we would be interested in the reason for the large hysteresis in the 6Fe and 8Fe alloys. Whether the product phase is the R-phase is further discussed in terms of the entropy change (Δ*S*).

**Figure 5:**
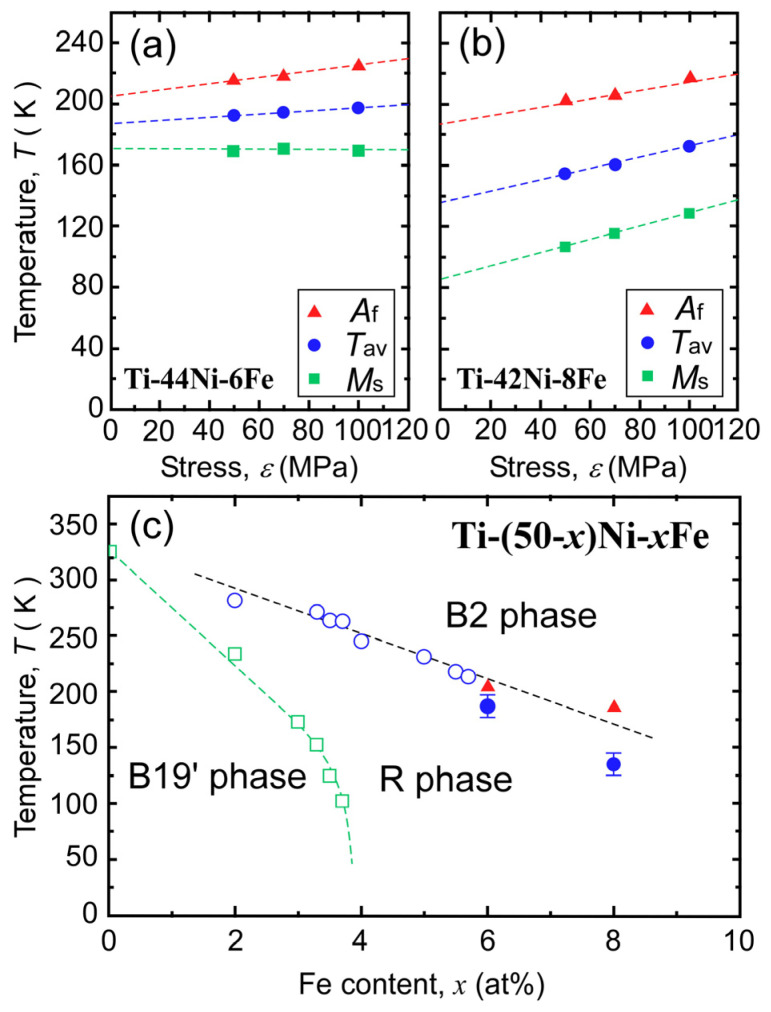
Stress dependence of *M*_s_, *A*_f_, and *T*_av_ of the Ti–44Ni–6Fe alloy (**a**) and those of the Ti–42Ni–8Fe alloys (**b**). *A*_f_, as plotted by solid red triangles, and *T*_av_ extrapolated to zero stress, as plotted by solid blue circles, are indicated in (**c**) together with the equilibrium temperature *T*_av_(R), as plotted by open blue circles, for the B2–R MT and *T*_av_(B19′), as plotted by open green squares, for the R-B19′ MT.

Figure 5 shows the *M*_s_ and *A*_f_ temperatures plotted as functions of the applied stress σ for the 6Fe alloy (a) and 8Fe alloy (b). The average *M*_s_ and *A*_f_, denoted as *T*_av_, were also plotted. *T*_av_ is frequently used as the experimentally obtained equilibrium temperature (*T*_0_) [42]. As an approximation, we consider *M*_s_, *A*_f_, and *T*_av_ to depend linearly on σ. Then, by extrapolating these values to σ = 0, we obtained *A*_f_ and *T*_av_ under zero stress, and the values are shown in phase diagram (c) by solid red triangles and solid blue circles, respectively. In the phase diagram, the equilibrium temperature of the B2–R MT (*T*_av_(R)) reported previously is shown by open blue circles, and that of R-B19′ MT is shown by open green squares [28].

The value of *A*_f_ lies on the extended line for *T*_av_(R), although *T*_av_ is slightly lower than the extended line. This result strongly suggests that the product formed in the 6Fe and 8Fe alloys under compressive stress was the R-phase.

**Figure 6:**
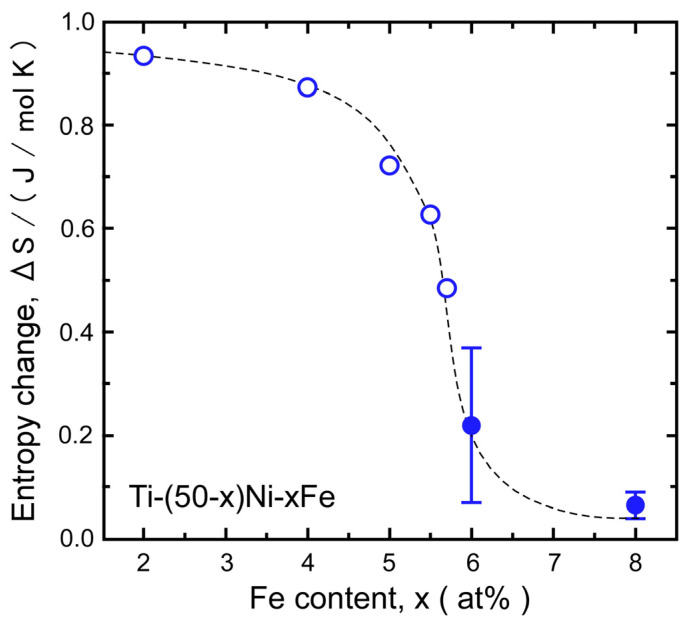
Entropy change for the B2–R transformation evaluated by the Clapeyron equation (solid circles). The value evaluated from the latent heat [28] is also shown by open circles. The dotted line is a visual guidance.

The B2–R MT described above is also supported by the entropy change in the MT. From the stress dependence of *T*_av_ shown in Figure 5, we evaluated the entropy change Δ*S* of the MT using the Clapeyron equation, given as d*T*/dσ = −Δ*ε*/Δ*S*. The transformation strain Δ*ε* was assumed to be 0.3%, as shown in Figure 2. The calculated value of Δ*S* is plotted as a function of the Fe content in Figure 6. The value of the B2–R MT evaluated previously [28] using DSC measurements is also shown. The Δ*S* values evaluated for the 6Fe and 8Fe alloys lie on the extended curve for the Δ*S* of the B2–R MT. This result again supports the conclusion that the product phase of the 6Fe and 8Fe alloys is the R-phase. Considering the X-ray diffraction results shown in Figure 4 and the discussion above, we can conclude that the product phase formed under compressive stress is the R-phase.

### 3.2. Reasons for Wide Temperature Hysteresis

Next, we discuss why the temperature hysteresis of the transformation exceeds 50 K, whereas the hysteresis for conventional B2–R MT is usually smaller than 5 K. As shown in Figure 5c, the thermally induced MT was suppressed when the iron content exceeded 6 at%, whereas the equilibrium temperature, *T*_av_(R), was expected to decrease linearly with the iron content, at least up to 8 at%. This implies that the B2–R MT was kinetically suppressed (thermally arrested). One important reason for this suppression is the insufficient driving force for the nucleation of the R-phase. As shown in Figure 2 and Figure 3, the B2–R MT of the 6Fe and 8Fe alloys were associated with a transformation strain of approximately 0.3%. A previous study [22], shows that the R-phase nucleates as a single variant. This implies that the elastic energy around the nuclei of the R-phase cannot be canceled by the self-accommodation of the variants. The driving force must overcome the increase in elastic energy caused by nucleation. The chemical driving force for the transformation can be approximated as the entropy change (Δ*S*) multiplied by supercooling (Δ*T*). As shown in Figure 6, the Δ*S* values of the 6Fe and 8Fe alloys were one order of magnitude smaller than that Δ*S* of the 2Fe alloy. This qualitatively explains why large supercooling is necessary for the 6Fe and 8Fe alloys.

The B2–R MT under [110]_B2_ compressive stress reported in this study was completely different from the MT under tensile stress in the [111]_B2_ direction, which we previously reported [40]. Under [111]_B2_ tensile stress, the strain increased gradually as the temperature decreased, and the hysteresis between the cooling and heating processes was less than 1 K. One possible reason for this difference is the MT path. Under [111]_B2_ tensile stress, one variant of the R-phase was preferable to the stress, whereas the remaining three variants were not. Subsequently, transformation could proceed without self-accommodation. The transformation between the B2-phase and a single variant of the R-phase may proceed continuously under stress, such as paramagnetic–ferromagnetic transition under a magnetic field. However, under [110]_B2_ compressive stress, two variants of the R-phase were preferable to the stress, whereas the remaining two variants were not. Thus, we expected self-accommodation to occur in two variants that are preferable to stress. In this case, a first-order transformation would be preferred because the lattice mismatch between the B2-phase and R-phase could be reduced by self-accommodation. Further investigation of the nucleation and growth of the R-phase variants is required in the future.

### 3.3. Kinetics of the B2–R Transformation

In a previous study [16], we reported that a 6Fe alloy exhibited isothermal B2–R MT when the specimen was held at 150 K. The isothermal nature of the B2–R MT implies that the thermal activation process assists the B2–R MT. The importance of the thermal activation process for the B2–R MT can be determined from the temperature dependence of the critical stress for the transformation. As shown in Figure 3, the critical stress for the B2–R MT increased as the temperature decreased. The increase in critical stress could be related to the reduction in thermal energy, which assists in the nucleation of the R-phase. Because kinetics plays an important role in the B2–R MT in 6Fe and the 8Fe alloys, the transformation depends strongly on the path. As shown in Figure 2, the 6Fe alloy was in the R-phase state at 110 K when cooled to 50 MPa. However, Figure 3 shows that the 6Fe alloy remains in the parent phase at 110 K under 50 MPa if the stress is applied after cooling to 110 K. As shown in Figure 3, the residual strain appeared at 150 and 110 K for the 6Fe alloy and at 120 and 100 K for the 8Fe alloy. The appearance of residual strain is explained by the phase equilibrium. As shown in Figure 5c, these temperatures are below *T*_av_, which can be regarded as the equilibrium temperature between the parent phase and R-phase. Therefore, the equilibrium state of the 6Fe and 8Fe alloys was in the R-phase at these temperatures, even under zero stress. Once the R-phase was induced with the assistance of compressive stress, it did not return to its parent phase. The specimen was heated above *A*_f_ to return to the B2-phase. By contrast, the residual strain did not appear in the stress–strain curves at 190 K (6Fe) and 150 K (8Fe), although an obvious hysteresis appeared at these temperatures. This indicates that a reverse transformation occurred at these temperatures. However, these temperatures were between *T*_av_ and *A*_f_. The internal stress around the R-phase likely assisted in the reverse transformation at these temperatures.

### 3.4. Relation between Softening and R-Phase Martensitic Transformation

**Figure 7:**
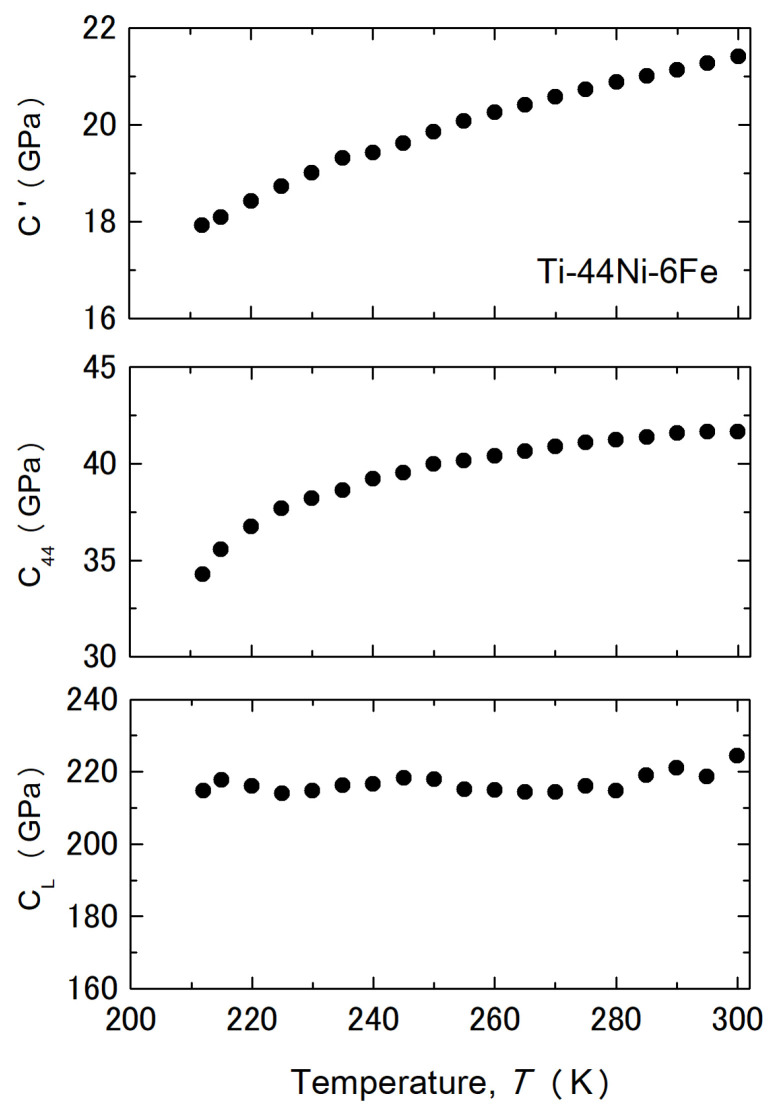
Temperature dependence of the elastic constants *C*′, *C*_44_, and *C*_L_ of the Ti–44Ni–6Fe alloy obtained by the RPR method.

In this section, we discuss the relationship between the lattice softening and the B2–R MT of the 6Fe and 8Fe alloys. Lattice softening is frequently regarded as a precursor to MT [21]. B2–R MT has also been discussed in relation to lattice softening [21,43,44,45,46]. Therefore, we studied the temperature dependence of the elastic constants of the 6Fe alloy using RPR. The resonance signal was very weak below 210 K, where the electrical resistivity was the smallest, and the appearance of diffuse satellites began, as shown in Figure 1. Therefore, only elastic constants were evaluated at temperatures above 210 K. The values of *C*′ = (*C*_11_ − *C*_12_)/2, *C*_44_, and *C*_L_ = (*C*_11_ + *C*_12_ + 2*C*_44_)/2 are shown in Figure 7.

**Figure 8:**
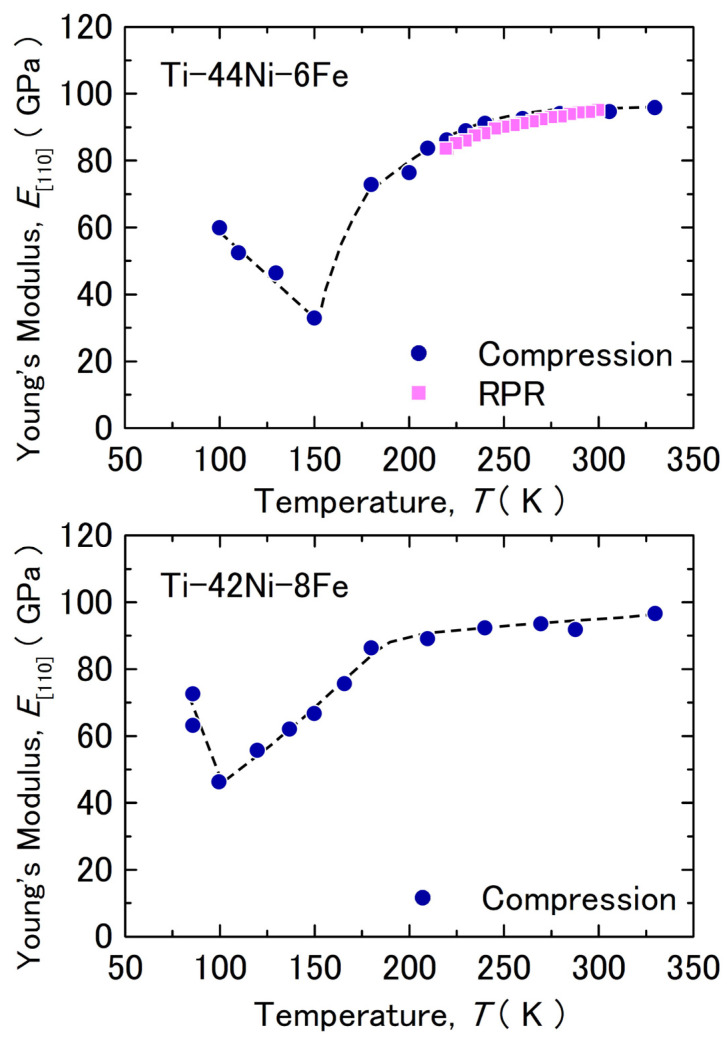
Temperature dependence of Young’s modulus in the [110]_B2_ direction obtained from the compressive test and that calculated from the elastic constants shown in Figure 7. The dotted line is for visual guidance.

Both *C*′ and *C*_44_ exhibited significant softening as the temperature decreased, whereas *C*_L_ was nearly independent of the temperature. This behavior resembles other Ti–Ni based alloys exhibiting a clear MT [43]. Figure 8 shows Young’s modulus in the [110]_B2_ direction evaluated from the initial slope of the stress–strain curves of the 6Fe and 8Fe alloys. The Young’s modulus evaluated from the elastic constants obtained by the RPR method is also shown by solid squares and was evaluated as follows [47]:

$$\frac{1}{E_{\left[110\right]}}=\frac{1}{9B}+\frac{1}{4C_{44}}+\frac{1}{6({C_{11}-C}_{44})}$$

where *B* is the bulk modulus, given by *B* = (*C*_11_ + *C*_12_)/3. The values evaluated by the stress–strain curve were consistent with those obtained using the RPR method. In both alloys, Young’s modulus decreased as the temperature decreased to ~150 K (6Fe) and ~100 K (8Fe) and then increased with a further decrease in temperature. We consider the fact that Young’s modulus shown in Figure 8 is that of the parent phase, because we used a slope below 10 MPa, which is sufficiently lower than the yield stress (critical stress) for the stress–induced MT. The *M*_s_ temperatures under a compressive stress of 50 MPa were ~170 K (6Fe) and ~110 K (8Fe), as shown in Figure 2. At these temperatures, Young’s modulus was approximately 50 GPa for both alloys. This value was nearly half of that at 300 K. This implies that the decrease in Young’s modulus is an important factor for the B2–R MT. Presumably, the decrease in the Young’s modulus reduces the driving force, which is necessary for the nucleation of the R-phase. The reduction in Young’s modulus is caused by the reduction in *C*′ and *C*_44_, as observed in Figure 7. We cannot yet confirm which one is more important for R-phase transformation, although the importance of *C*′ is frequently regarded as a precursor phenomenon of MTs [43,44,45,46]. The absolute value of *C*′ was smaller than that of *C*_44_ in the examined temperature range, but *C*_44_ showed a sharp decrease below 240 K. Thus, the value of *C*_44_ could be the same as *C*′ near 150 K in the 6Fe alloy. An evaluation of the elastic constants below 210 K is necessary for further discussion.

Incidentally, Young’s modulus in the [111] direction (*E*_[111]_) of the 6Fe alloy decreases continuously as the temperature decreases to 100 K [40]. This behavior is entirely different from the increase in *E*_[110]_ observed at temperatures below 150 K (Figure 8). Considering the fact that *E*_[111]_ is approximated as 1/*E*_[111]_~1/3*C*_44_, we consider the increase in *E*_[110]_ below 150 K to have been caused by an increase in *C*′. This temperature also blunted the change in the position of the diffuse satellites, which may have been related to the growth of the nanoscale domain microstructure.

## 4. Conclusions

Although the Ti–44Ni–6Fe and Ti–42Ni–8Fe alloys do not show conventional thermally induced B2–R MT in the absence of external stress, they show MT under compressive stress applied in the [110]_B2_ direction. Likely, B2–R MT was kinetically arrested in these alloys. MT is characterized by a temperature hysteresis larger than 50 K. The insufficient driving force caused by the low entropy change for the B2–R MT in these alloys is the primary reason for the kinetic arrest. The softening of the elastic constants likely assists the B2–R MT.

## Data Availability

Data are contained within the article.
